# Shifts in Symbiotic Endophyte Communities of a Foundational Salt Marsh Grass following Oil Exposure from the Deepwater Horizon Oil Spill

**DOI:** 10.1371/journal.pone.0122378

**Published:** 2015-04-29

**Authors:** Demetra Kandalepas, Michael J. Blum, Sunshine A. Van Bael

**Affiliations:** 1 Department of Ecology and Evolutionary Biology, Tulane University, New Orleans, Louisiana, United States of America; 2 Tulane-Xavier Center for Bioenvironmental Research, Tulane University, New Orleans, Louisiana, United States of America; Umeå Plant Science Centre, Umeå University, SWEDEN

## Abstract

Symbiotic associations can be disrupted by disturbance or by changing environmental conditions. Endophytes are fungal and bacterial symbionts of plants that can affect performance. As in more widely known symbioses, acute or chronic stressor exposure might trigger disassociation of endophytes from host plants. We tested this hypothesis by examining the effects of oil exposure following the Deepwater Horizon (DWH) oil spill on endophyte diversity and abundance in *Spartina alterniflora* – the foundational plant in northern Gulf coast salt marshes affected by the spill. We compared bacterial and fungal endophytes isolated from plants in reference areas to isolates from plants collected in areas with residual oil that has persisted for more than three years after the DWH spill. DNA sequence-based estimates showed that oil exposure shifted endophyte diversity and community structure. Plants from oiled areas exhibited near total loss of leaf fungal endophytes. Root fungal endophytes exhibited a more modest decline and little change was observed in endophytic bacterial diversity or abundance, though a shift towards hydrocarbon metabolizers was found in plants from oiled sites. These results show that plant-endophyte symbioses can be disrupted by stressor exposure, and indicate that symbiont community disassembly in marsh plants is an enduring outcome of the DWH spill.

## Introduction

Disturbance or shifts in environmental conditions can disrupt widespread and often obligate symbiotic associations. Many symbionts confer benefits to hosts, such as nutrient acquisition (*e*.*g*., mycorrhizae and nitrogen-fixing bacteria), protection through production of toxins (*e*.*g*., cyanobacteria in cycads), and production of photosynthate (*e*.*g*., algae in lichens and zooxanthellae in corals). Although these relationships are often beneficial to both partners, changes in environmental conditions, such as those brought on by climate change or acute disturbances, may result in disassociation. For example, corals expel zooxanthellae when ocean temperature spikes [1, 2], pH drops [3, 4], or when salinity increases [5]. Disruption of symbiotic associations may place a temporary strain on the host, or in some cases lead to host death. Consequently, the loss of symbionts can propagate to population and community-wide mortality, which in turn can result in sustained loss of biodiversity and ecosystem services [6–9].

Endophytes are microorganisms that live asymptomatically within plant tissue [10]. These symbionts have been found throughout the plant kingdom and have been described as “hyperdiverse” [11]. Endophytes are increasingly becoming recognized as contributors to plant performance. Some endophytic bacteria are known to directly and indirectly protect host plants against pathogens [12–15] and others increase nutrient acquisition [16]. Fungal endophytes have been shown to increase host tolerance to biotic stress, including pathogen and herbivore damage [17–19], but also to abiotic stress, such as drought, high salinity, and extreme temperatures [20, 21]. For example, Redman *et al*. [22] were able confer salt and drought tolerance to rice plants through inoculation with specific salt- and temperature-adapted endophytes. Additionally, endophytes have been shown to increase plant growth, water retention, and chlorophyll concentration, as well as protect hosts against oxidative stress [23]. Though the influence of endophytes can drive host plant demography [24] and shape plant communities [25–27], and though several studies have demonstrated mutualistic relationships between endophytes and plants, little is known about endophyte responses and subsequent effects on plant hosts following stressor exposure [28].

Prior studies suggest that endophyte community structure shifts in response to stressor exposure. Some specialized endophyte species have been shown to thrive under extreme stress, such as hypersaline coastal ecosystems and high temperatures from hydrothermal vents [20]. Symbioses with plants in these cases have been described as “habitat-adapted symbiosis,” indicating that each partner in the symbiosis requires the presence of the other to survive under such conditions [22]. Kandalepas [29] showed, however, that endophyte diversity in the obligate marsh plant *Sagittaria lancifolia* decreases and that endophyte composition changes with chronic salt and flood stress. This suggests that exposure to stress may lead to sustained loss of endophytes and disassociation of endophyte-host plant symbioses.

**Fig 1:**
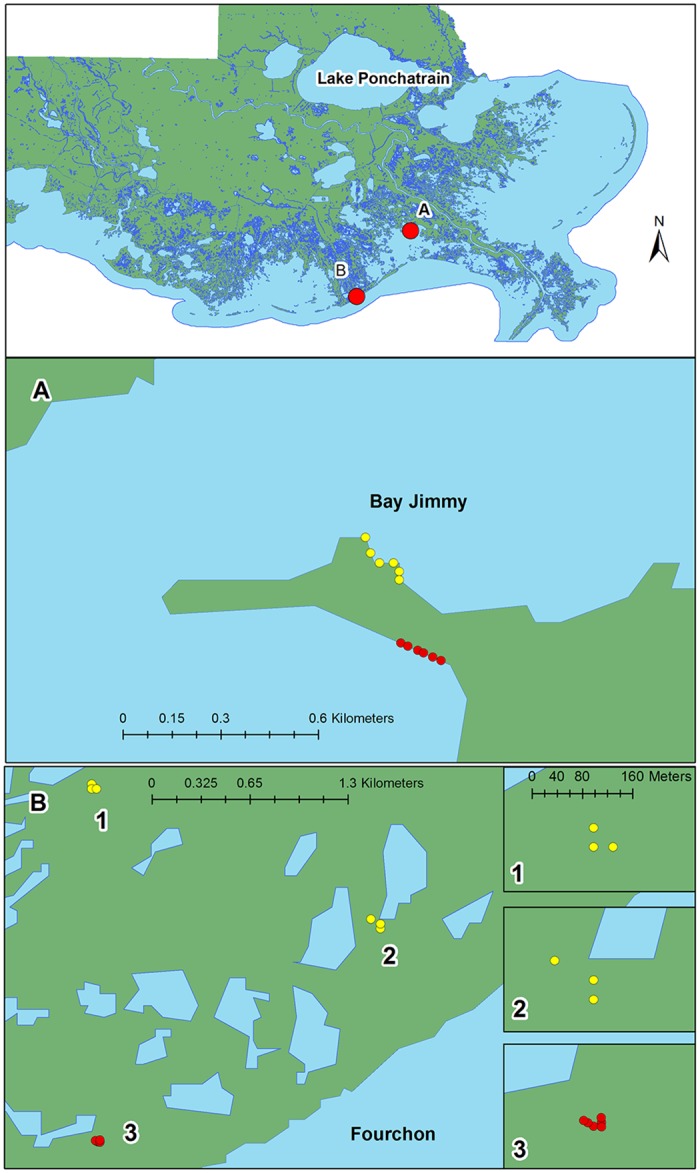
Map of study area. (A) The location of *S*. *alterniflora* collections for oiled (yellow circles) and unoiled reference (red circles) areas at Bay Jimmy; and (B) the location of *S*. *alterniflora* collections for oiled and unoiled reference areas at Fourchon. Specific GPS coordinates for each area at both sites are provided in Table A in S1 File. Images were obtained from the Louisiana Oil Spill Coordinator's Office (LOSCO), 20000120, Louisiana Land and Water Interface, Geographic NAD83, LOSCO (2000) (landwater_interface_LOSCO_1992). Metadata for these maps are available at http://lagic.lsu.edu/data/losco/landwater_interface_losco_1992.html.

Greater understanding of endophyte responses to stress could shed light on fundamental ecological processes and offer novel perspective on how symbioses can sustain at-risk ecosystems. In this study, we examine the effects of oil exposure on endophytes of *Spartina alterniflora* (smooth cordgrass)—a foundational species of salt marshes across the Atlantic and Gulf coasts of North America. Smooth cordgrass inhabits high saline environments that are frequently flooded—stressful conditions that exceed the tolerance limits of nearly all plant species [30]. Prior studies suggest that other salt marsh plants harbor a high diversity and abundance of endophytes that shift following exposure to recurring natural abiotic stress [31, 29]. Here we examined endophytes from *S*. *alterniflora* plants in marshes that were oiled by the Deepwater Horizon (DWH) oil spill in 2010, as well as from plants in nearby reference areas that were not oiled (Fig 1). Our objective was to determine whether stressor exposure can result in sustained loss of endophytes in a system where elevated susceptibility of plant hosts to stress or injury could cascade into further reinforcing loss of endophyte diversity, host plant integrity, and ecosystem function [32]. We hypothesized that exposure to novel stress might result in pronounced shifts in endophyte community composition, including sustained loss from plant hosts. Fungal and bacterial endophytes isolated from roots and leaves were examined to determine whether common endophytes disassociate from plant hosts following oil exposure, while hydrocarbon-metabolizing fungi and bacteria persist [33]. Evidence of either outcome would suggest that the structure and composition of endophyte communities could serve as a new class of biological indicators of stressor exposure, including hydrocarbon contamination, in coastal marshes and other at-risk ecosystems.

## Methods

### Site location and sampling

We obtained samples in November and December of 2013 from two sites in southeast Louisiana: a marsh in Bay Jimmy in Northern Barataria Bay (Fig 1A; Table A in S1 File) and a second marsh behind Fourchon Beach, which lies in front of adjoining Caminada Bay (Fig 1B; Table A in S1 File). The Bay Jimmy site is a salt marsh island of predominantly organic soil with some clay content. The vegetation at the site consists primarily of *Spartina alterniflora*, though *Distichlis spicata* and *Salicornia virginica* are found on the shoreline. The Fourchon Beach site is a salt marsh overlying predominantly organic soil with some sand content. The site is dominated by *Spartina alterniflora*, *Salicornia virginica*, and *Avicennia germinans*. Despite extensive remediation and cleanup efforts, the persistence of residual surface and subsurface oil indicates that resident biota remain susceptible to exposure at both sites [34–36].

Paired oiled and control samples were collected at each study site. We collected oiled samples on the southwest shore of the Bay Jimmy marsh island (Fig 1A), which experienced some of the heaviest accumulations of oil from the DWH spill [34, 36]. We collected control plants on the northwestern shoreline of the same island (Fig 1A). Though no oil was observed on this shoreline in September 2010 [34], Zengel and Michel [34] reported evidence of oiling in September 2011. We did not observe oil on the shoreline at the time we obtained plants for this study, and we used a hexane:acetone 50:50 v/v indicator to confirm that oil was not present at the time of collection. At Fourchon Beach, we collected samples along a transect crossing a heavily oiled to lightly oiled gradient parallel to the marsh edge, where oil deposits at the time of collections were concentrated at the southwest end of the transect (Fig 1B). We obtained control plants from the Fourchon site approximately 1 km northeast of the oiled site (Fig 1B) in an area dominated by *Spartina alterniflora*. Oil has never been observed in this area [34].

We harvested twelve individual *Spartina alterniflora* Loisel. (Poaceae) plants from each study site—six from each oiled area and six from each reference area. We gently washed roots in seawater to remove agglomerated soil and oil on site. Plants were transported in cold storage and then refrigerated upon arrival to Tulane University. Roots and leaves were processed for endophytes within 24 to 36 hours of collecting.

### Root endophyte culturing

We removed 10 to 15 apparently healthy roots close to the main stem of each plant. Each root was cut into 3 to 5 cm pieces. We then rinsed the cut roots in de-ionized (DI) water three times to remove any residual soil and oil. We surface-sterilized roots in 70% ethanol for 10 seconds, 50% bleach for 2 minutes, followed by three rinses in sterile DI water [37]. After surface sterilization, we aseptically removed 5mm from each end of each root segment. We split the remaining root segments into two groups for (1) staining and microscopy and (2) culturing. The roots stained for microscopy were immediately placed in sterile 50 ml falcon tubes and stored in 50% ethanol until further processing. We cut the root segments for culturing into 5 mm segments under aseptic conditions.

For each plant collected from oiled areas, we plated root segments onto four 15 mm petri dishes—two containing 2% malt extract agar (MEA; 2%, BD Bacto malt extract), which selects for fungi, and two containing nutrient media (Oxoid recipe as per manufacturer guidelines), which selects for bacteria [37]. We plated roots from control plant samples on only one plate per plant per media type, as pilot sampling recovered approximately an order of magnitude more endophyte isolates from reference relative to oiled sites [38]. We used six root segments on each plate to avoid contamination among endophytes. We incubated root tissue segments in the dark at room temperature (24°C) and isolated new colonies on to fresh media to create axenic cultures. Once sufficient growth had occurred, we categorized cultures into morphospecies according to visual characters. Morphospecies assignments were subsequently confirmed with Sanger sequencing.

### Leaf endophyte culturing

We removed four to five healthy, green leaves from each plant. The leaves were rinsed in tap water for 1 min and then sliced into 2 mm x 2 mm sections. We immersed the sections with a tea strainer in 95% ethanol for 10 sec, followed by 2 min in 10% bleach, then 2 min in 70% ethanol [11]. For each oiled plant, 16 randomly selected sections were plated on 2% MEA and nutrient agar (two plates per media type). As with root segments, we plated leaf sections from reference-area plants on to one plate per media type because of greater isolation rates. We incubated the leaf tissue segments in ambient conditions away from direct sunlight. New growths were then isolated, grown in pure cultures, and classified to morphospecies. All morphospecies designations were confirmed with Sanger sequencing.

### Sanger sequencing of morphospecies

We sequenced up to 15 isolates of each morphospecies assignment. Total genomic DNA was extracted from each of the selected pure cultures using a Qiagen DNAeasy Plant Mini Kit following the TissueLyser protocol with the exception that fresh tissue was used. We used genomic DNA in polymerase chain reaction (PCR) mixtures following Hoffman and Arnold [38]. Each 25 ml PCR reaction mixture included 12.5 μl Sigma REDTaq (Sigma Aldrich; 20 mM Tris-HCl, pH 8.3, 100 mM KCl, 3 mM MgCl_2_, 0.002% gelatin, 0.4 mM mixed dNTPs, stabilizers, 0.06 units Taq polymerase / ml.), 0.4 μM of each primer, 10–25 ng DNA template, and 9.5 μl of PCR-quality water [38]. For fungal morphospecies, we amplified the nuclear ribosomal internal transcribed spacer (nrITS) and partial large subunit (LSU) using primers ITS1F (5'-CTTGGTCAT TTAGAGGAAGTAA) or ITS5 (5'-GGAAGTAAAAGTCGTAACAAGG) for the forward reaction and LR3 (5'-GGTCCGTGTTTCAAGAC) or ITS4 (5'-TCCTCCGCTTATTGATATGC) for the reverse reaction (http://lutzonilab.org/16s-ribosomal-dna/). Our cycling protocol for amplification followed Arnold and Lutzoni [39]. The same protocol was followed for bacterial morphospecies, except we amplified the 16S rDNA gene using the universal primers 27F (5'-AGAGTTTGATCMTGGCTCAG) and 1492RI (5'-GGTTACCTTGT TACGACTT).

PCR products were confirmed by visualization with SYBR on 1% agarose gels, and submitted to Beckman Coulter Genomics (Boston, MA) for Sanger sequencing. Sequence assembly and editing were carried out with Sequencher v5.0 (Gene Codes Corp., Ann Arbor, MI) with support from Mesquite [40]. Sequences with a minimum 97% similarity were considered to be representative of the same operational taxonomic unit (OTU) [40]. Representative sequences of each OTU were compared to NCBI archives through BLAST searches to assign putative taxonomic identities based on sequence similarity and query coverage (Table 1, Table B in S1 File). Voucher cultures of all OTUs were archived in the Van Bael lab at Tulane University, under accession numbers 431–946. Accession numbers for sequences deposited in the NCBI Genbank database are KP757431-KP757718.

**Table 1:** Endophyte operational taxonomic units (OTUs) that predominated in isolates from *S*. *alterniflora* roots and leaves, with putative taxonomic assignments (NCBI Genbank accession numbers, % identity and % sequence cover given in Table B of S1 File).

OTU with putative taxonomic assignment	Kingdom	Average Abundance* OILED	Average Abundance UNOILED REFERENCE	Percent Contribution^§^	Cumulative Percent Contribution^¶^
**BAY JIMMY ROOTS**					
*Bacillus pumilus*	Eubacteria	1.0	1.44	12.40	12.40
*Lulworthia purpurea*	Fungi	0.33	0.44	6.65	19.05
*Magnaporthe oryzae*	Fungi	0.33	0.22	4.70	23.76
*Talaromyces helices*	Fungi	0.17	0.44	4.68	28.44
*Mangrovibacter plantisponsor*	Eubacteria	0.17	0.33	4.68	33.12
*Lulworthia* sp.	Fungi	0.33	0.22	4.55	37.67
*Bacillus cereus*	Eubacteria	0.50	0.11	4.49	42.16
*Pseudomonas* sp.	Eubacteria	0.33	0.11	4.48	46.64
*Phaeosphaeria* sp. 3	Fungi	0	0.44	4.47	51.11
*Pseudallescheria* sp.	Fungi	0.33	0.00	4.01	55.12
*Achromobacter marplantensis*	Eubacteria	0.17	0.22	3.78	58.90
*Phaeosphaeria* sp. 2	Fungi	0.33	0.00	3.07	61.97
*Microsphaeropsis arundinis*	Fungi	0.17	0.11	2.66	64.64
Unknown Hypocreales	Fungi	0.17	0.11	2.66	67.30
Unknown Pleosporales	Fungi	0	0.22	2.51	69.81
**BAY JIMMY LEAVES**					
*Phaeosphaeria* sp. 2					
*Bacillus pumilus*	Fungi	0.40	1.75	22.14	22.14
*Achromobacter marplantensis*	Eubacteria	1.80	0.50	21.04	43.18
*Bacillus* sp.	Eubacteria	0	1.13	9.38	52.56
*Xanthomonas* sp.	Eubacteria	0.4	0.13	9.32	61.88
*Pseudoxanthomonas spadix*	Eubacteria	0	0.25	4.59	66.47
FOURCHON ROOTS					
*Escherichia hermannii*	Eubacteria	0	1.22	15.74	15.74
*Vibrio* sp.	Eubacteria	0.67	1.00	12.51	28.25
*Bacillus pumilus*	Eubacteria	0.67	0.33	9.89	38.13
*Phaeosphaeria* sp. 3	Fungi	0	1.00	9.59	47.73
*Pseudomonas* sp.	Eubacteria	0	0.78	8.40	56.13
*Phaeosphaeria* sp. 2	Fungi	0	0.56	6.82	62.94
*Lulworthia purpurea*	Fungi	0	0.44	6.30	69.24
**FOURCHON LEAVES**					
*Phaeosphaeria* sp. 2	Fungi	1	4.38	74.22	74.22
*Bacillus pumilus*	Eubacteria	0	0.25	5.40	79.62

*Average abundance is presented for each OTU for oiled and reference areas. Average abundance was calculated using SIMPER in PRIMER v.7. All abundance values for each OTU within a group, (*e*.*g*., all abundance values for *Bacillus pumilus* within Bay Jimmy Roots from oiled areas) were summed and this total divided by the number of samples within the group.

^§^ Percent contribution is a measure of the amount of variation explained by each OTU separately within each group (Bay Jimmy Roots).

^¶^ Cumulative percent contribution is the running total variation accounted for by a given set of OTUs within a group. For example, the first four OTUs listed within the Bay Jimmy roots group, together account for 28.44% of the total variation within this group, and the sixteen OTUs listed for this group account for 72.04% of the total variation within Bay Jimmy roots.

### Data analysis

We generated rarefaction curves using EstimateS [41] based on the OTUs obtained from sequence alignments. Curves were generated for the entire data set, as well as for all oiled and all unoiled reference samples. To estimate diversity, we calculated Shannon Diversity based on the OTUs recovered for each oiled and unoiled reference site. Using Systat 13 (Systat Software, Inc., San Jose, CA, USA), we conducted ANOVAs to test for differences in overall, fungal, and bacterial diversity between sites (Bay Jimmy versus Fourchon), between treatments (oiled versus unoiled reference), and between tissue types (roots versus leaves) accounting for all two- and three-way interactions. We also conducted ANOVAs to test for differences in abundance. Furthermore, we examined whether community composition differed between treatments by carrying out ordinations using nonmetric multidimensional scaling (NMS) [42]. We determined whether groups (site, oil regime, plant tissue type—leaves or roots) significantly affected community separation among samples using analysis of similarity (ANOSIM) [42] in PRIMER v7, beta version [43]. We used the similarity percentages (SIMPER) [42] routine in PRIMER v7 to identify OTUs driving differences in community composition. We used a Distance-Based Linear Model (DistLM) [44, 45] to determine the genera that significantly contributed to the total solution. DistLM is a non-parametric permutational routine used to model linear relationships within a multivariate cloud [45]. Finally, we used Canonical Analysis of Principle Coordinates to clarify the groups determined in NMS.

### Oil culture experiment

We supported our field collection data with *in vitro* comparisons of endophyte growth on petri plates with and without crude oil. After autoclaving nutrient agar, we added 1% unrefined crude oil (Unrefined Texas Crude Oil, www.skajaquoda.com) and 0.25% Tween 80, using a 0.2 micron syringe filter. The oiled plates received 1% crude oil and Tween 80, while the control plates received an addition of Tween 80 but no crude oil. We plated 4 mm plugs from 51 distinct field-collected bacterial and fungal endophytes, placing each isolate onto one oiled and one control plate. All plates were stored together at room temperature (~27°C). We photographed each pair of plates every 2 days until the cultures were 14 days old. We used ImageJ (http://imagej.nih.gov/ij/) to trace and calculate the area of each culture on the photos from day 14. The area of each colony on day 14 was used to calculate percent change [(oil_area_—control_area_) / control_area_) *100] in colony size among pairs. For five strains that grew rapidly, we used photos from day 4 to calculate the percent increase or decrease.

To test for an overall effect of oil on culture growth, we used the absolute values of percent change in a one-sample t-test comparing colony means to zero (*i*.*e*., zero reflects no change in colony size with respect to oil). We also used general linear models to assess variation in percent change of colony size with respect to symbiont type (bacterium or fungus), symbiont site provenance (oiled or unoiled site), and symbiont substrate provenance (leaf or root). Our models initially included symbiont type, site, substrate, and all interactions, but we removed site provenance after finding a non-significant effect, which suggests that endophyte responses to oil exposure are not contingent on host plant condition or that *in vitro* assays minimize potential legacies of exposure response. The final model tested the effect of symbiont type, substrate, and their interaction on percent change of colony size.

## Results

### Effects of oil contamination on endophyte community composition

**Fig 2:**
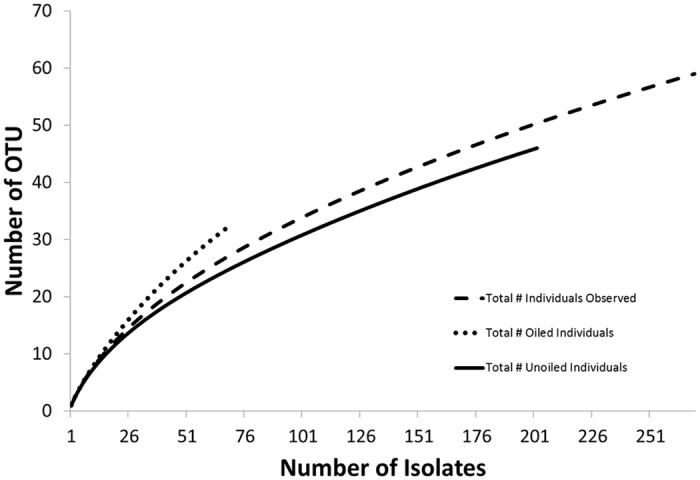
Rarefaction curves for the number of operational taxonomic units (OTUs) by group. The dotted line corresponds to samples from oiled areas, the solid line represents samples from unoiled reference areas, and the dashed line corresponds to all samples combined.

We obtained 507 bacterial and fungal endophyte isolates from leaves and roots of *S*. *alterniflora* plants from oiled and unoiled reference sites in Louisiana (Fig 1). We acquired DNA sequences from a subset of 271 isolates, which represented 60 OTUs (Fig 2), for which we could assign to 41 genera, 32 families, 23 orders, 13 classes, 8 phyla, and three kingdoms. A Distance-Based Linear Model (DistLM) revealed that the genera that contributed significantly (>5% of the solution and p<0.05) to our solution were: *Bacillus* (Eubacteria; 34.6%; F_7,33_ = 2.91, p = 0.001), *Phaeosphaeria* (Fungi; 21.92%; F_11,29_ = 3.66, p = 0.001), *Lulworthia* (Fungi; 13.91%; F_13,27_ = 6.36, p = 0.001), and *Vibrio* (Eubacteria; 8.09%; F_16,24_ = 3.02, p = 0.006). Approximately 83% of the variation in our data set is attributable to these genera.

**Fig 3:**
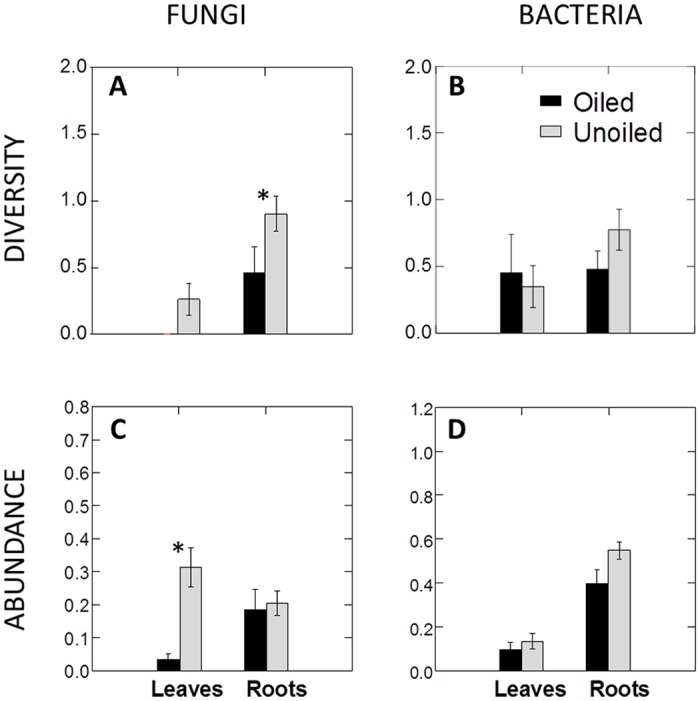
Linear contrasts for foliar and root endophyte diversity and abundance according to treatment. (A) Fungal endophyte diversity in leaves and roots; (B) bacterial endophyte diversity in leaves and roots; (C) fungal endophyte abundance in leaves and roots; and (D) bacterial endophyte abundance in leaves and roots. Black bars correspond to oiled areas and grey bars correspond to unoiled reference areas. * = significant differences. Error bars are standard errors.

We observed significantly lower endophyte diversity and abundance in plants from oiled versus unoiled reference areas. Fungi appeared to be more sensitive to oil contamination than bacteria. Fungal endophyte diversity was significantly lower in leaves (p<0.001) and roots (p = 0.024) from plants exposed to oil (Fig 3A). Foliar fungal endophytes were nearly absent in plants from oiled areas (Fig 3A and 3C; p<0.001). Oiling did not influence the abundance of root fungal endophytes. The diversity of foliar and root bacterial endophytes was similar in plants from oiled and unoiled reference areas (Fig 3B). Bacterial endophyte abundance also did not differ in either tissue type with oil contamination (Fig 3D).

**Fig 4:**
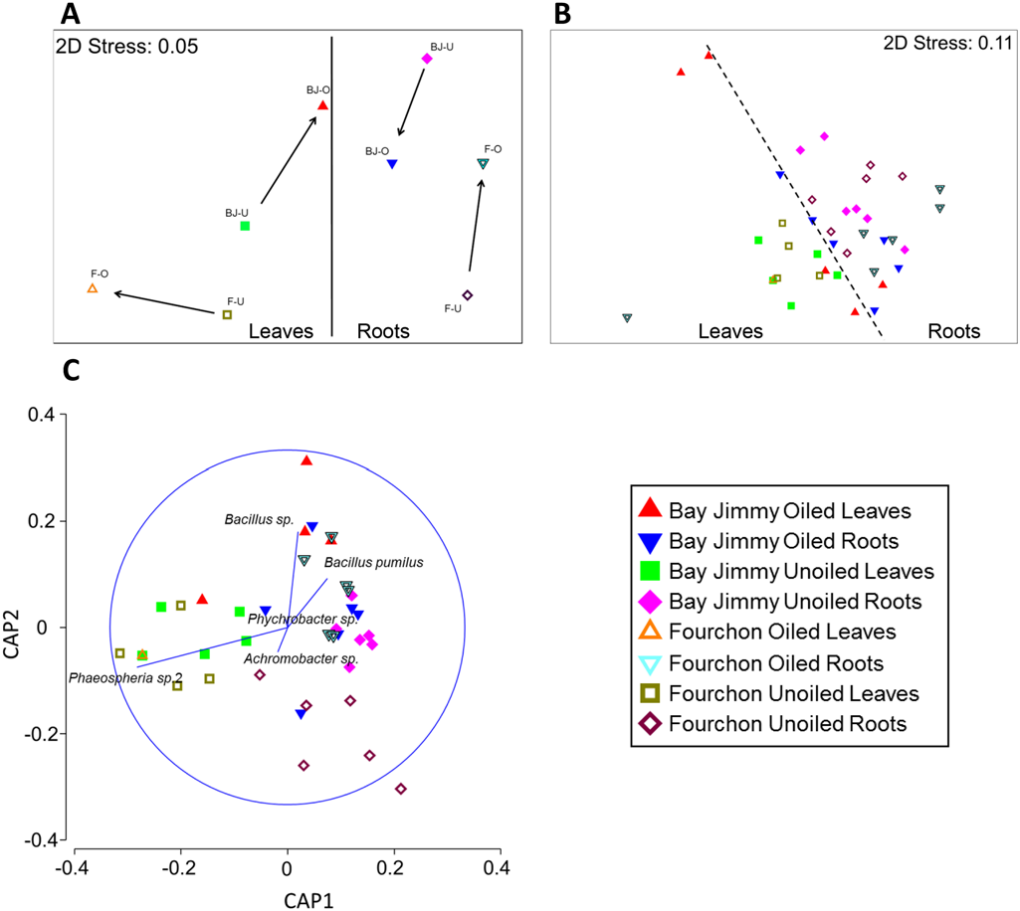
Ordinations of endophyte community data. (A) Nonmetric multidimensional scaling using centroids for foliar and root communities from oiled and unoiled reference areas in Bay Jimmy and Fourchon. Arrows indicate the direction of community change between oiled and unoiled reference conditions. (B) Canonical Analysis of Principal Coordinates on unconstrained data illustrating community differences according to study location, oiling and tissue type with superimposed vectors of OTUs driving community differences.

Variation in endophyte community structure was related to tissue type, sampling location (Bay Jimmy or Fourchon, Fig 1), and presence or absence of oil. The structure of root and leaf endophyte communities was significantly different (Fig 4; ANOSIM R = 0.45, p = 0.001). Furthermore, the communities found in the two tissue types exhibited distinct responses to oil exposure. Foliar endophyte communities in plants from unoiled reference areas were similar, and oil exposure resulted in divergent shifts in community structure (Fig 4A, Figure A in S1 File). Conversely, root endophyte communities from unoiled reference areas in the two study locations were dissimilar, and oil exposure resulted in convergent shifts in root endophyte community structure (Fig 4A, Figure B in S1 File).

A two-dimensional ordination of all the samples confirmed that the strongest shift in community composition is attributable to tissue type (Fig 4B). Further community compositional differences are attributable to exposure regime (*i*.*e*., oiled versus unoiled reference areas) nested within tissue type, and location. For example, we observed distinct root endophyte communities between plants from oiled and unoiled reference areas within study sites (ANOSIM: R = 0.2; p = 0.017). We also detected distinct root endophyte communities between study sites (ANOSIM: R = 0.196; p = 0.006). Low isolation frequency from plants sampled from oiled areas prevented us from drawing quantitative comparisons of foliar endophyte structure at the Fourchon study site. However, as was observed in the comparison of root communities, the foliar endophyte communities from plants in unoiled reference areas were distinct from the foliar communities in plants from oiled areas at Bay Jimmy (Fig 4, ANOSIM: R = 0.44; p = 0.009).

Similar patterns of endophyte community shifts arising from oil exposure were identified through Canonical Analysis of Principal Coordinates (CAP, Fig 4C). Root endophyte communities from plants in unoiled reference areas were, for example, strongly divergent from root communities in plants from oiled areas. A more pronounced shift was found in comparisons within the Fourchon site relative to the Bay Jimmy site. The CAP analysis also showed a strong separation of foliar endophyte community composition between plants from oiled and unoiled reference areas within Bay Jimmy. A vector overlay of endophyte OTUs (Table 1) driving the observed community shifts (Fig 4C) indicates that *Bacillus* bacteria are more strongly associated with plants in oiled areas, while fungi in the genus *Phaeosphaeria* are more common in plants at unoiled reference areas (Fig 4C).

### Effects of oil exposure on endophyte growth

**Fig 5:**
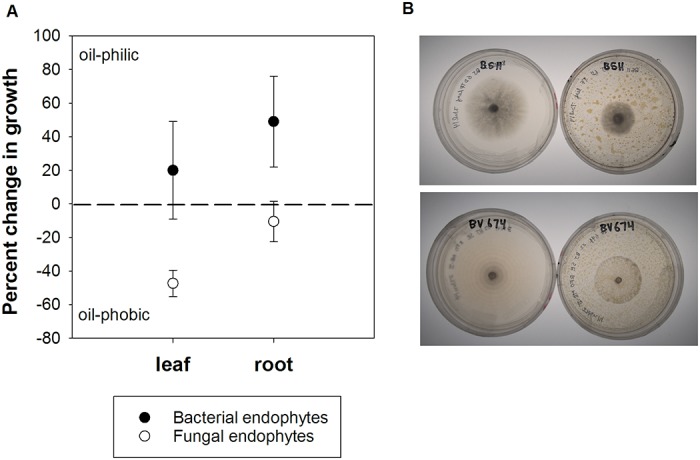
The results of *in vitro* growth assays showed that oil reduced the growth of some endophyte strains, but increased growth for others. (A) Mean (±1 standard error) percent change in colony growth shows that bacteria tended to be more oil-philic while fungi were relatively oil-phobic. The dotted zero line represents no difference in growth on oiled versus unoiled media. (B) The top panel shows growth differences for an oil-phobic fungus isolated from a leaf, while the bottom panel shows an oil-phobic fungus isolated from a root.

Assays of growth in media with and without crude oil confirmed that fungal and bacterial endophytes differentially respond to oil exposure (Fig 5). Endophyte isolates exhibited positive or negative growth in the presence of oil. Out of 51 culture assays, 31 endophyte isolates exhibited slower growth in the presence of oil (oil-phobic; median percent change in growth was -39%) while 20 endophyte isolates exhibited faster growth (oil-philic; median percent change in growth was 63%). Overall, the mean percent change in growth of the colonies on oiled versus control plates was negative (-18%), with the absolute percent change significantly different from zero (*i*.*e*. zero means no effect of oil on growth; t = 24.9, d.f. = 50, p<0.001). Bacteria tended to be more oil-philic whereas fungi tended to be more oil-phobic (Fig 5; symbiont type; F_1,47_ = 6.16, p = 0.017). There was no significant effect of host plant provenance (*i*.*e*., whether the plant was sampled from oiled or unoiled reference sites) on the percent change in growth of different endophyte cultures. Symbionts isolated from leaves tended to be more oil-phobic than those from roots but the difference was not significant (Fig 5; substrate provenance; F_1,47_ = 1.81, p = 0.19).

## Discussion

Like other widespread symbionts, endophyte communities can be disrupted by stressor exposure. Our finding that oil contamination altered root and foliar endophyte communities within a foundational salt marsh plant demonstrates that the principle of anthropogenic disruption of host-symbiont interactions [3, 4] extends to plant-endophyte systems. Our results also illustrate that the DWH spill has left a lasting imprint on the ecology of northern Gulf of Mexico shorelines. We detected pronounced shifts in fungal and bacterial endophyte communities within *S*. *alterniflora* from oiled shorelines sampled three years after the spill. The diversity and abundance of endophytes were markedly lower in plants from oiled areas, largely reflecting the loss of foliar endophytic fungi. The composition of remnant communities also reflected shifts toward oil-philic OTUs, which mostly consisted of oil-philic bacteria. Though the role of endophytes in *S*. *alterniflora* remains undetermined, the disruption of endophyte community structure could debilitate host plants through the loss of stress tolerance or other key physiological functions [20, 22, 46, 47]. *Spartina alterniflora* can achieve full vegetative recovery in as little as seven months after 100% oil cover [48], but our findings indicate that full ecological and physiological function may not be recovered years after initial exposure. Thus, even if vegetative regrowth is achieved following an oiling event, *S*. *alterniflora* may be more susceptible to other stressors, such as inundation and nutrient loading, which are linked to marsh loss [49–52]. If so, then the duration and magnitude of direct impacts attributable to the DWH spill may be amplified by cascading vulnerability arising from the disruption of ecological interactions.

### Endophyte abundance and diversity

Foliar fungal endophytes were nearly absent in plants from oiled areas. This contrasts sharply with evidence that fungi became dominant constituents of metazoan communities in beach sediments oiled during the DWH spill [33]. Our growth experiments demonstrated that the observed loss could be an outcome of oil toxicity, but oiling may also indirectly shape foliar fungal endophyte communities. The foliar endophytes examined in this study are horizontally transmitted via spores dispersed via air currents. While outside of the host plant tissue, endophyte spores are susceptible to environmental influences that may decrease inoculum potential. Spore density or viability on plant surfaces or other substrates could be reduced if oiling prevents endophytes from penetrating leaf surfaces or extending hyphal growth within tissues of oiled plants. Physiological responses of host plants to oil exposure also may shape endophyte community structure. For example, plant uptake of PAHs [53] might select for endophytes that are able to metabolize hydrocarbons and derivative by-products inside of plant tissues [54]. Though further work will be necessary to test this hypothesis, our survey results support the possibility of functional retention of select endophytes in stressed host plants. Additional support for this comes from evidence of elevated concentrations of PAHs on leaf surfaces, in the cuticle, as well as within the leaf tissue of *S*. *alterniflora* concurrently sampled from oiled areas at Bay Jimmy [55].

More modest responses were detected for root fungal endophytes and bacterial endophytes. We observed lower fungal endophyte diversity in roots from plants sampled in oiled areas, but in contrast to foliar fungal endophytes, oiling did not influence the abundance of root fungal endophytes. No differences in bacterial endophyte diversity or abundance were detected. Evidence of reduced root fungal endophyte diversity is consistent with evidence of lower fungal diversity in beach sediments oiled by the DWH spill [33]. The loss of fungal diversity in beach sediments has been attributed to oil-induced environmental stress favoring resilient, opportunistic species able to capitalize on the availability of new resources [33]. Our findings suggest that the observed shifts in fungal endophyte communities may be attributable to a similar process, though oiling can also influence the availability of host-derived carbon and nutrients, reduce inoculum potential, and prevent penetration of root tissue. Discriminating among these possibilities will require examining the *S*. *alterniflora* rhizosphere to determine the prevalence of fungal spores typical of an endophytic lifestyle. Bacterial endophytes appear to be less sensitive to oiling than fungal endophytes, but it is also possible that bacterial endophyte responses are more transient than fungal responses. Oiling can elicit punctuated and temporary shifts in bacterial communities. Beazley *et al*. [56] found, for example, that the relative abundance and number of bacterial OTUs increased sharply in oiled salt marsh sediments during the DWH spill, reflecting a spike in the abundance of hydrocarbon-degrading microbial populations. Our findings do not exclude the possibility that bacterial endophyte communities underwent rapid succession, possibly related to the use of different hydrocarbon compound classes [57]. Indeed, our surveys and growth assays suggest that oil exposure elicited the rise of hydrocarbon-degrading OTUs, resulting in lasting shifts in the composition of bacterial endophyte communities.

### Community structure

Oil exposure altered endophyte community structure in *S*. *alterniflora*, with contrasting pathways of response in foliar versus root communities. Oiling resulted in the divergence of foliar communities and the convergence of root communities. The observed response trajectories are likely a reflection of selective retention acting in conjunction with dispersal pathways through aerial and subsurface environments. Foliar endophytes tend to be wind dispersed as spores, while root endophytes are largely transported by water in soil [58]. Therefore greater similarity would be expected for foliar communities among our unoiled reference areas, as dispersal is likely not limiting across the spatial scale of this study. Stressor exposure, however, may limit the potential of spore inoculum potential, thereby increasing the possibility that the community structure of foliar endophytes reflects the intensity and persistence of oil within the local environment. Conversely, root endophytes would be expected to be less similar in unoiled reference areas, as heterogeneous soil conditions and dispersal limitation are known to affect subsurface microbial community structure [58]. Thus community dissimilarity is likely a reflection of stochastic and limited dispersal promoting ecological drift [59]. Convergence may subsequently result from oiling acting as a selective filter via toxicity, effects on inoculum potential, penetration of root tissue, or resource availability.

The composition of bacterial endophyte communities in plants sampled from oiled areas was similar to bacterial communities found in salt marsh sediments and beach sands oiled during the DWH spill [56, 57, 60, 61]. Nearly 40% of the bacterial endophytes found in plants from oiled areas belonged to the phylum *Proteobacteria*, with most *Proteobacteria* OTUs identified as *Alpha*- and *Gammaproteobacteria*. Many of these bacteria have been linked to degradation of recalcitrant hydrocarbons, including PAHs, in contaminated sediments [62–64]. The most common bacterial endophyte in our study, however, was *Bacillus pumilus* (Phylum: *Firmicutes*), a nitrogen fixing bacterium [65] prevalent in high salinity environments [66] known to promote plant growth and protect roots and leaves from fungal pathogens [66–69]. *Firmicutes* are known to contain several hydrocarbon-metabolizing families, including *Bacillaceae* [56]. *Bacillus pumilus* was equally or more abundant in samples from oiled versus unoiled reference areas, except in leaf tissues taken from oiled areas at Fourchon, in which it was altogether absent. Beazley *et al*. [56] similarly found that abundance of *Firmicutes* in oiled salt marsh sediments steadily increased during and after the oil spill, even when oil was no longer detectable. As community enrichment of *Firmicutes* has also been observed in other affected coastal environments, including oiled beaches [70], the group could serve as an indicator of hydrocarbon degradation, particularly when more recalcitrant compounds like PAHs are present.

Though comparably little is known about the function of constituent members of fungal communities, our findings indicate that the observed shifts are attributable to differential responses of indigenous taxa to the presence of oil. The most common fungal strain found in our study was a species of *Phaeosphaeria*. We found that this strain was more prevalent in roots and leaves from plants sampled in Fourchon, as well as in leaves from plants sampled in Bay Jimmy, but it was absent in roots from plants collected in Bay Jimmy. This discrepancy could reflect functional variation among *Phaeosphaeria* lineages, variation in length of time since oil exposure, or under sampling. *Phaeosphaeria* are known to be one of the primary decomposers in salt marshes dominated by *S*. *alterniflora* [71]. *Phaeosphaeria* fungi also are being explored as potential agents for bioremediation of oil contamination [72]. Members of this group are known to contain lignolytic laccase enzymes, which have been shown to aid in the detoxification of organic pollutants within the rhizosphere [73]. Evidence of elevated production of these enzymes in oiled marsh sediments also has been attributed to hydrocarbon degradation arising from plant-microbe interactions [56].

The observed shifts in endophyte structure may reflect underlying responses to stressor exposure. Many of the families found in our study are known to promote the integrity of host plants through other mechanisms. For example, *Bacillus cereus*—which was found in roots from plants in oiled areas of Bay Jimmy—can promote root and shoot growth as well as higher chlorophyll concentrations [74, 23]. *Bacillus taquilensis* can protect plants from oxidative stress [23]. *Bacillus cereus* and *B*. *taquilensis* also can increase water retention, thereby ameliorating salt stress in host plants [23]. Though *Phaeosphaeria* is a promising tool for bioremediation [72], our findings suggest that these fungi may influence a range of functions. The dominant strain detected within *S*. *alterniflora* appears to be systemic, as it was isolated from leaves and roots. Thus endophytes may serve dual or multiple purposes, and some may exhibit different functions when host plants are exposed to novel conditions like oil contamination. Further study is warranted to determine the functional plasticity of endophytes and to better understand the importance of individual versus community-level contributions to host plant survival and fitness.

More intensive sampling and a wider range of assays could offer further understanding of *in vitro* responses to exposure, and *in planta* experiments could clarify how *in vitro* assays reflect *in planta* conditions. Because a great majority of bacteria and fungal endophytes are not culturable, use of culture independent approaches (e.g., genomic assays) could also enrich understanding of taxonomic and functional responses of endophyte communities to stress and disturbance.

## Conclusions

Our findings indicate that stressor exposure can result in sustained loss of endophytes in a system where elevated vulnerability of plant hosts could cascade into functional deficits that reshape whole ecosystems. Reduced integrity of *S*. *alterniflora* hosts could, for example, translate to lower growth and productivity, which in turn could reduce accretion and increase erosion [50]. Further work will be necessary to determine organismal and ecosystem outcomes of endophyte loss in *S*. *alterniflora*. Additionally, further work will be necessary to determine the pace and trajectory of endophyte community reassembly following stressor exposure. Our findings suggest that the presence of oil impedes the reassembly of endophyte communities in *S*. *alterniflora*, with rates of reassembly contingent on composition (*i*.*e*., bacterial versus fungi), and tissue type (*i*.*e*., leaf versus root). It is also likely that the duration of stressor exposure (*i*.*e*., acute versus chronic) also drives reassembly rates. Identifying the conditions that control reassembly would not only aid in rapid assessment of stressor exposure and ecological integrity [57], it would also promote the development of methods to facilitate reconstitution of symbioses and novel approaches to accelerate recovery of at-risk ecosystems. For example, resilience might be enhanced by inoculating host plants with indigenous endophytes [22] or protective benefits might be conferred with targeted endophytes known to metabolize contaminants [75] which could lead to more effective remediation and ecosystem restoration.

## Supporting Information

S1 File Supplementary results, figures, GPS coordinates and NCBI Genbank Accession numbers for Table 1, including %identity and %coverage for top matches. (DOCX)
