# Supplementary material for: Shifts in Symbiotic Endophyte Communities of a Foundational Salt Marsh Grass following Oil Exposure from the Deepwater Horizon Oil Spill
Source: PLoS One. 2015 Apr 29;10(4):e0122378. doi: 10.1371/journal.pone.0122378 (PMC4414556; doi:10.1371/journal.pone.0122378)
Supplement: S1 File — (DOCX) [file pone.0122378.s001.docx]

**S1**

**SUPPORTING INFORMATION**

**Supplementary Results**

**Overall endophyte diversity**

Overall endophyte diversity differed according to site, oiling, and tissue type (root versus leaves) (Site: F_1,96_=10.112, p=0.002; Oiling: F_1,96_=6.101, p=0.015; Tissue: F_1,96_=25.792, p<0.001). Bacteria were not more diverse, overall, than fungi. Endophytes were more diverse at Bay Jimmy than Fourchon, and communities in reference areas were more diverse than communities in oiled areas. Endophytes also were more diverse in roots than in leaves. Additionally, we detected a significant interaction between site and oil regime (F_1,96_=8.228, p=0.005). Reference areas were more diverse than oiled areas at Fourchon (F_1,96_=5.354, P=0.023), but there was no difference in endophyte diversity between oiled and reference areas at Bay Jimmy. A marginally significant 3-way interaction was found among site, oil regime, and tissue (F_1,96_=3.614, p=0.06). Endophytes were more diverse in roots from plants sampled in reference (F_1,96_=4.757, p=0.032) and oiled areas (F_1,96_=8.436, p=0.005) at Bay Jimmy, as well as in plants from reference areas at Fourchon (F_1,96_=20.183, p<0.001). There was no difference in endophyte diversity between roots and leaves in plants from the oiled area at Fourchon.

**Fungal endophyte diversity**

Fungal diversity differed according to site, oiling, and tissue (Site: F_1,34_=4.703, p=0.037; Oiling: F_1,34_=6.924, p=0.013; Tissue: F_1,34_=16.051, p<0.001). Fungi were more diverse in Bay Jimmy than Fourchon. Reference areas harbored greater diversity than did oiled areas. Fungi also were more diverse in roots than in leaves. A marginally significant interaction was detected for site and tissue (F_1,34_=3.574, p=0.067); more diverse fungal community were found in roots from plants sampled at Bay Jimmy (F_1,36_=16.772, p<0.001), whereas fungal diversity did not differ between roots and leaves of plants sampled at Fourchon. A marginally significant 3-way interaction also was detected (F_1,34_=3.868, p=0.057). Roots harbored a greater diversity of endophytes in oiled (F_1,32_=15.219, p<0.01) and reference areas (F_1,32_=7.516, p=0.01) at Bay Jimmy, as well as in reference areas at Fourchon (F_1,32_=7.148, p=0.012). Fungal diversity did not differ between roots and leaves of plants sampled from the oiled area at Fourchon.

**Bacterial endophyte diversity**

Though bacterial diversity predominately differed according to tissue type (F_1,33_=5.673, p=0.023), some differences were attributable to site and oil regime. Bacterial endophytes were more diverse in roots than in leaves. No other main effect was significant, but a significant interaction was detected between site and oil regime (F_1,33_=6.687, p=0.014). The reference area at Fourchon had greater bacterial endophyte diversity than did the oiled area (F_1,36_=4.364, p=0.044), whereas no difference was detected between the reference and oiled areas at Bay Jimmy. A significant interaction also was detected for site and tissue (F_1,33_=5.152, p=0.030). Bacterial endophytes were more diverse in roots than in leaves of plants from Fourchon (F_1,36_=5.339, p=0.027). Bacterial diversity did not differ between roots and leaves of plants from Bay Jimmy.

**Overall endophyte abundance**

Overall endophyte abundance differed according to oiling, tissue, and symbiont category (Oiling: F_1,107_=16.784, p<0.001; Tissue: F_1,107_=38.469, p<0.001; Symbiont: F_1,107_=12.813, p=0.001). Endophytes were more abundant in reference than oiled areas. Greater abundance also was detected in roots than in leaves, and bacteria were more abundant than fungi. A significant interaction was found between site and oil regime (F_1,107_=4.198; p=0.043). Endophytes were more abundant in reference than in oiled areas at Fourchon, whereas no difference was detected between reference and oiled areas at Bay Jimmy. A marginally significant interaction was detected between site and tissue (F_1,107_=3.902, p=0.051). Endophytes were more abundant in roots than in leaves from plants sampled at Bay Jimmy (F_1,116_=57.615, p<0.001), but not from plants sampled at Fourchon. A significant interaction also was detected between site and symbiont (F_1,107_=4.703, p=0.032); bacteria were more abundant than fungi at Bay Jimmy (F_1,104_=18.528, p<0.001) but not at Fourchon. Additionally, a significant interaction was detected between tissue and symbiont (F_1,107_=31.759, p<0.001). Bacteria were more abundant than fungi in roots (F_1,104_=46.940, p<0.001), whereas no difference occurred in leaves. A marginally significant interaction was detected between site, oil regime, and symbiont (F_1,107_=3.392, p=0.068). Bacteria were more abundant than fungi in reference (F_1,104_=15.52, p<0.001) and oiled areas (F_1,104_=5.477, p=0.021) at Bay Jimmy, as well as in oiled area at Fourchon (F_1,104_=4.122, p=0.045), but no difference was found in the reference areas at Fourchon. Finally, we detected a significant interaction between oil regime, tissue, and symbiont (F_1,107_=9.836, p=0.002). Fungi were more abundant than bacteria in leaves from plants sampled from reference areas (F_1,104_=12.138, p<0.001), whereas bacteria were more abundant in roots from plants sampled from oiled (F_1,104_=11.36, p=0.001) and reference areas (F_1,104_=44.955, p=0.001). Fungi and bacterial abundance did not differ in leaves from oiled areas.

**Fungal endophyte abundance**

Fungal endophyte abundance predominantly differed according to oiling, though some variation was attributable to differences among sites and tissues. The abundance of fungal endophytes was higher in reference than in oiled areas (F_1,52_=13.845, p<0.001). We also detected a significant interaction between site and oil regime (F_1,52_=8.335, p=0.006). Linear contrasts revealed that fungal endophyte abundance was higher in the reference areas than the oiled area at Fourchon (F_1,52_=21.832, p<0.001), but not at Bay Jimmy. Additionally, we detected a significant interaction between oil regime and tissue (F_1,52_=10.438, p=0.002); the abundance of fungi was marginally higher in leaves than in roots of plants sampled from reference areas (F_1,56_=3.159, p=0.081) whereas fungal abundance was higher in roots than in leaves of plants sampled from oiled areas (F_1,56_=3.973, p=0.051). Another significant interaction was detected between site and tissue (F_1,52_=13.005, p=0.001). Fungi were more abundant in roots than in leaves of plants at Bay Jimmy (F_1,52_=8.465, p=0.005), whereas the opposite was found at Fourchon (F_1,52_=4.798, p=0.033). Finally, a marginally significant 3-way interaction was detected (F_1,52_=3.753, p=0.058). Fungi were more abundant in leaves than in roots of plants from reference areas at Fourchon (F_1,52_=21.352, p<0.001). There was no difference between leaves and roots in reference areas at Bay Jimmy, but fungi were more abundant in roots than in leaves of plants from oiled areas at Bay Jimmy (F_1,52_=6.093, p=0.017). Fungal abundance did not differ between roots and leaves of plants from the oiled area at Fourchon.

**Bacterial endophyte abundance**

Bacterial endophyte abundance differed according to site, oiling, and tissue (Site: F_1,55_=7.060, p=0.01; Oil regime: F_1,55_=5.584, p=0.022; Tissue: F_1,52_=78.983, p<0.001). Bacterial abundance was greater at Bay Jimmy than at Fourchon, and greater abundance was also observed in reference areas than in oiled areas. Bacterial abundance also was greater in roots than in leaves. We did not detecte any significant interactions.

**Community composition in leaves and roots**

To examine the patterns shown in our complete data set in more detail, we analyzed foliar and root endophyte community composition separately (Figures A and B). ANOSIM showed that leaf endophyte communities differed significantly between oiled and reference host plant samples (R= 0.44; p=0.009), as well as between study sites (R=0.143; p=0.045). NMS showed a strong separation between study sites (Figure A), and CAP showed that, within Bay Jimmy, there was additional separation between oiled and reference host plant samples (Figure A). The most common OTUs associated with these samples are presented as vectors within Figure A and listed in Table 1. Based on SIMPER, the OTUs that drove this solution were primarily bacteria, however the fungus *Phaeosphaeria sp.* 2 was the most common endophyte OTU in reference host plant samples for both study sites. This OTU was rarely found in the oiled host plant samples. *Phaeosphaeria sp.* 2 accounted for 24.6% of the variation in Bay Jimmy samples and 73.4% of the variation in the Fourchon samples. Together with *Bacillus pumilus*, the two OTUs accounted for 43.3% and 81.7% in Bay Jimmy and Fourchon respectively. Other OTUs that contributed to the variation in Bay Jimmy were *Achromobacter marplatensis* (15%), another *Bacillus sp.* (7.5%), and *Psychrobacter alimintarius* (4.7%). ANOSIM showed that, within roots, there were significant differences in endophyte community composition between the two study sites (R=0.22; p=0.006) and marginally significant differences between oiled and reference host plant samples (R=0.152; p=0.06). NMS showed some separation between sites and between oiled and unoiled samples within Fourchon (Figure B). CAP further separated the samples by site, as well as by oil regime, though the latter was most pronounced within Fourchon (Figure B). The reference host plant samples from Bay Jimmy were characterized by different endophyte OTUs than reference samples from Fourchon (Table 1). The root endophyte communities from Bay Jimmy were composed primarily of fungi, whereas the Fourchon samples contained mostly bacteria. The endophyte OTUs that were dominant in roots from host plants sampled in Bay Jimmy according to SIMPER were: *Bacillus pumilus* (bacterium; unoiled reference; 12.4%), *Lulworthia purpurea* (fungus; reference; 6.65%), *Magnaporthe oryzae* (fungus; oiled; 4.7%), and *Talaromyces helices* (fungus; unoiled reference; 4.68%). At Fourchon, root endophyte communities were dominated by: *Escherichia hermannii* (bacterium; unoiled reference; 15.74%), *Vibrio* sp*.* (bacterium; unoiled reference; 12.51%), *B. pumilus* (bacterium; oiled; 9.98%), *Phaeosphaeria* sp*.* 3 (fungus; unoiled reference; 9.59%), *Pseudomonas* sp*.* (bacterium; unoiled reference; 8.4%), *Phaeosphaeria* sp*.* 2 (fungus; unoiled reference; 6.82%), and *Lulworthia purpurea* (fungus; unoiled reference; 6.3%).


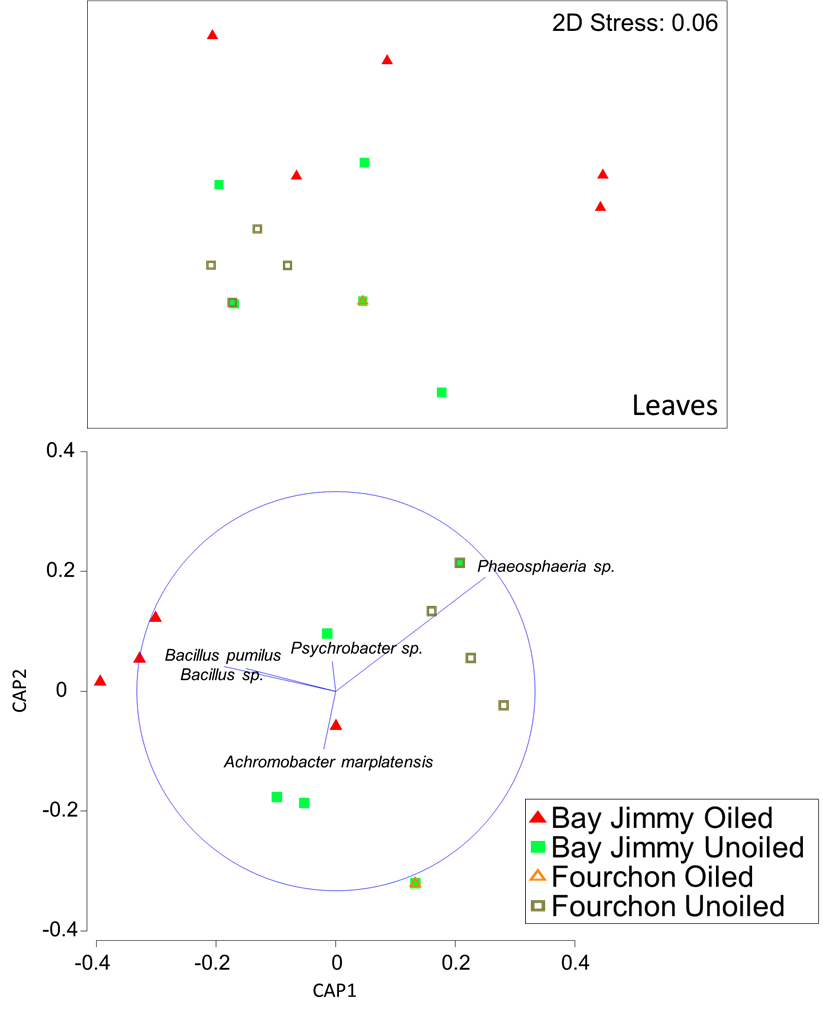


**Figure A.** Ordinations of leaf endophytes. (A) NMS of endophyte communities from oiled and reference hosts from Bay Jimmy and Fourchon. (B) CAP analysis showing further separation of foliar endophyte communities from oiled and unoiled reference hosts from Bay Jimmy, with superimposed vectors of OTUs driving community differences. **Red triangles** = leaves from Bay Jimmy oiled area; **green squares** = leaves from Bay Jimmy unoiled reference area; **open orange triangle** = leaves from Fourchon oiled area; **open green squares** = leaves from Fourchon unoiled reference areas.

**B**

**A**


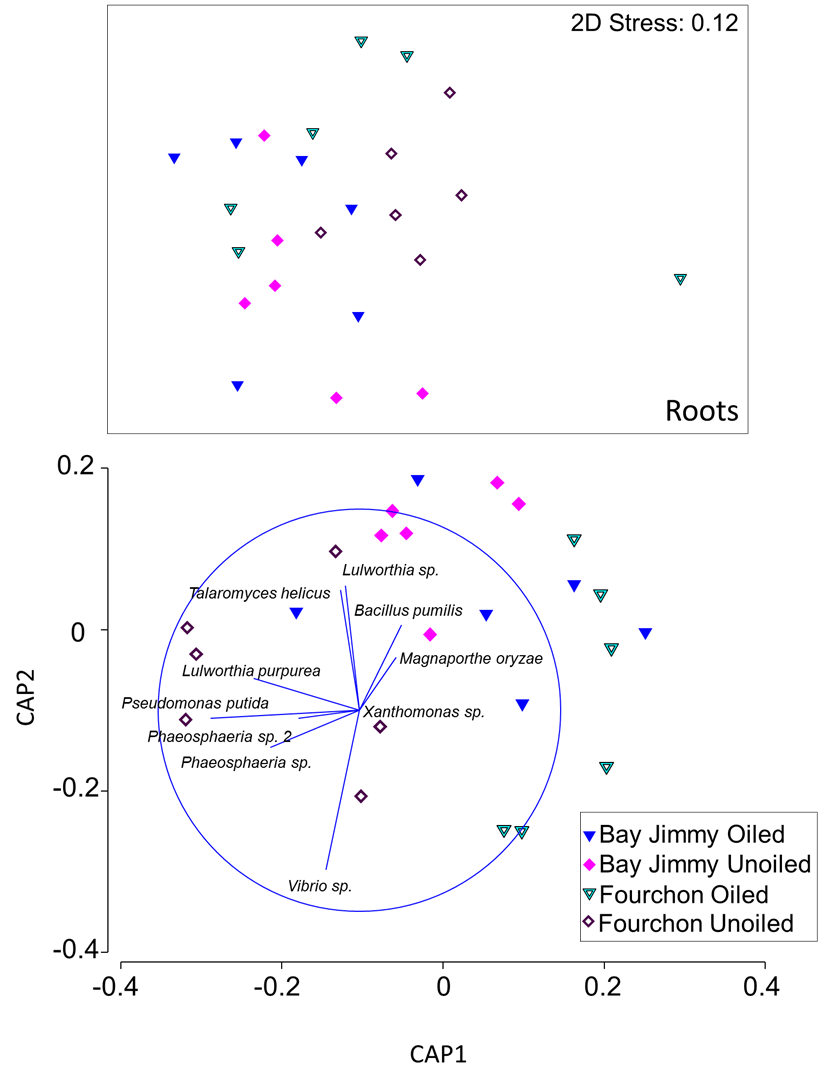
**Figure B.** Ordinations of root endophytes. (A) NMS of endophyte communities from plants sampled in oiled and reference areas at Bay Jimmy and Fourchon. (B) CAP analysis showing further separation of root endophyte communities from oiled and unoiled reference areas within Fourchon and within Bay Jimmy, with superimposed vectors of OTUs driving community differences. **Blue triangles** = roots from Bay Jimmy oiled areas; **pink diamonds** = roots from Bay Jimmy unoiled reference areas; **open green triangle** = roots from Fourchon oiled area; **open diamonds =** roots from Fourchon unoiled reference areas.

**B**

**A**

**Table A.** GPS Coordinates for samples collected from oiled and reference areas at both study sites.

|  |  | GPS Coordinates | |
| --- | --- | --- | --- |
| Site | Oil Regime | Latitude | Longitude |
| Bay Jimmy | Oiled | 29°26’37.66”N | 89°53’14.74”W |
|  |  | 29°26’37.43”N | 89°53’14.20”W |
|  |  | 29°26’36.92”N | 89°53’13.56”W |
|  |  | 29°26’36.52”N | 89°53’12.83”W |
|  |  | 29°26’36.13”N | 89°53’12.30”W |
|  |  | 29°26’35.64”N | 89°53’11.67”W |
|  | Reference | 29°26’43.32”N | 89°53’21.45”W |
|  |  | 29°26’42.87”N | 89°53’20.94”W |
|  |  | 29°26’42.75”N | 89°53’20.44”W |
|  |  | 29°26’42.46”N | 89°53’19.83”W |
|  |  | 29°26’41.86”N | 89°53’19.24”W |
|  |  | 29°26’41.32”N | 89°53’19.06”W |
| Fourchon | Oiled | 29°8’0.16”N | 90°8’43.65”W |
|  |  | 29°8’0.33”N | 90°8’43.94”W |
|  |  | 29°8’0.46”N | 90°8’44.13”W |
|  |  | 29°8’0.13”N | 90°8’43.24”W |
|  |  | 29°8’0.38”N | 90°8’43.26”W |
|  |  | 29°8’0.59”N | 90°8’43.30”W |
|  | Reference | 29°9’16.44”N | 90°8’44.43”W |
|  |  | 29°9’16.25”N | 90°8’45.34”W |
|  |  | 29°9’17.02”N | 90°8’44.59”W |
|  |  | 29°8’46.40”N | 90°7’43.04”W |
|  |  | 29°8’46.72”N | 90°7’42.75”W |
|  |  | 29°8’46.68”N | 90°7’43.41”W |

**Table B.** Operational Taxonomic Units (OTUs) from Table 1, with information from the NCBI GenBank and sequence accession numbers from our study.

| OTU with putative taxonomic assignment | Kingdom | | | Accession  Number for match | % Identity | % Query Cover | Accession number for our sequence |  |
| --- | --- | --- | --- | --- | --- | --- | --- | --- |
| **BAY JIMMY ROOTS** |  | | |  |  |  |  |  |
| *Bacillus pumilus* | Eubacteria | | | KC815305 | 100 | 100 | KP757583 |  |
| *Lulworthia purpurea* | Fungi | | | JN886815 | 96 | 50 | KP757515 |  |
| *Magnaporthe oryzae* | Fungi | | | JQ747492 | 93 | 92 | KP757446 |  |
| *Talaromyces helices* | Fungi | | | AF033396 | 99 | 99 | KP757497 |  |
| *Magrovibacter plantisponsor* | Eubacteria | | | KJ604963 | 99 | 99 | KP757590 |  |
| *Lulworthia* sp*.* | Fungi | | | JN886815 | 99 | 51 | KP757526 |  |
| *Bacillus cereus* | Eubacteria | | | KJ801578 | 100 | 100 | KP757622 |  |
| *Pseudomonas* sp. | Eubacteria | | | JQ658418 | 100 | 99 | KP757671 |  |
| *Phaeosphaeria* sp. 3 | Fungi | | | JF449819 | 94 | 87 | KP757543 |  |
| *Pseudallescheria* sp. | Fungi | | | FJ345358 | 100 | 84 | KP757447 |  |
| *Achromobacter marplantensis* | Eubacteria | | | KF876913 | 100 | 100 | KP757604 |  |
| *Phaeosphaeria* sp. 2 | Fungi | | | JF449819 | 95 | 92 | KP757493 |  |
| *Microsphaeropsis arundinis* | Fungi | | | EF094556 | 99 | 100 | KP757564 |  |
| Unknown Hypocreales | Fungi | | | KJ812343 | 100 | 50 | KP757571 |  |
| Unknown Pleosporales | Fungi | | | KC248543 | 93 | 87 | KP757569 |  |
| *Marinomonas* sp. | Eubacteria | | | JF710987 | 99 | 99 | KP757618 |  |
|  |  | | |  |  |  |  |  |
| **BAY JIMMY LEAVES** |  | | |  |  |  |  |  |
| *Phaeosphaeria* sp. 2 | Fungi | | | JQ759472 | 95 | 92 | KP757432 |  |
| *Bacillus pumilus* | Eubacteria | | | KJ482902 | 100 | 100 | KP757686 |  |
| *Achromobacter marplantensis* | Eubacteria | | | KF876913 | 100 | 100 | KP757601 |  |
| *Bacillus* sp. | Eubacteria | | | KF170316 | 100 | 100 | KP757690 |  |
| *Xanthomonas* sp. | Eubacteria | | | EU313793 | 100 | 99 | KP757709 |  |
| *Pseudoxanthomonas spadix* | Eubacteria | | | NR042580 | 99 | 99 | KP757644 |  |
|  |  | | |  |  |  |  |  |
| **FOURCHON ROOTS** |  | |  | | | | | |
| *Escherichia hermannii* | | Eubacteria | | JX968500 | 100 | 100 | KP757588 |  |
| *Vibrio* sp. | | Eubacteria | | KJ877669 | 100 | 99 | KP757617 |  |
| *Bacillus pumilus* | | Eubacteria | | KJ620763 | 100 | 100 | KP757581 |  |
| *Phaeosphaeria* sp. 3 | | Fungi | | KC966352 | 94 | 87 | KP757489 |  |
| *Pseudomonas* sp. | | Eubacteria | | HM461889 | 100 | 100 | KP757587 |  |
| *Phaeosphaeria* sp. 2 | | Fungi | | JQ759472 | 95 | 92 | KP757438 |  |
| *Lulworthia purpurea* | | Fungi | | JN886815 | 99 | 50 | KP757520 |  |
|  | |  | |  |  |  |  |  |
| **FOURCHON LEAVES** | |  | |  |  |  |  |  |
| *Phaeosphaeria* sp. 2 | | Fungi | | JQ759472 | 95 | 92 | KP757503 |  |
| *Bacillus pumilus* | | Eubacteria | | EU231626 | 100 | 100 | KP757708 |  |
|  | |  | |  |  |  |  |  |
